# A novel method to determine the antiviral efficacy of hand rubs using murine norovirus (MNV) as surrogate of human norovirus

**DOI:** 10.1186/1753-6561-5-S6-P27

**Published:** 2011-06-29

**Authors:** G Kampf, E Steinmann, J Steinmann

**Affiliations:** 1 Scientific Affairs, Bode Chemie GmbH, Hamburg, Germany; 2 Division of Experimental Virology, TWINCORE, Hannover, Germany; 3 Mikrolab GmbH, Bremen, Germany

## Introduction / objectives

So far there is no method available in Europe to determine the antiviral efficacy on contaminated hands. Aim was therefore to develop a method which resembles both contamination and hand treatment in clinical practice as closely as possible.

## Methods

Each fingerpad (8 fingers per 4 subjects) was contaminated by dipping for 15 s in 0.4 ml of MNV suspension + 10% stool suspension and allowed to dry. The virus titre on fingers was determined by shaking a plastic vial with 1 ml of sampling fluid for 20 s. Infectivity was determined by transferring 100 µl after dilution to 8 wells of a microtitre plate with 100 µl of *RAW 264.7 cells*. After 4 days cultures were assessed for cytopathic effects, the infective dose was calculated with the method of Spearman and Kärber. Hands were similar to EN 1500 either treated with 3 ml of a hand rub or with 3 ml of water of standard hardness using responsible application. Four hand rubs (based on ethanol 80%, 85% or 95%, or based on a combination of 30% propan-1-ol, 45% propan-2-ol and 0.2% mecetronium etilsulphate) and one hand gel (based on 85% ethanol) were evaluated.

## Results

Mean baseline viral titre was between 5.99 and 6.52. The ethanol-based products reduced the viral load by 4.32 ± 0.69 (80% ethanol), 4.59 ± 0.36 (85% ethanol, rinse), 4.52 ± 0.67 (85% ethanol, gel) and 4.44 ± 1.07 (95% ethanol) which were all significantly more effective compared to the application of water (means between 2.29 ± 0.45 and 2.67 ± 0.61). The propanol-based hand rub was somewhat less effective (3.77 ± 0.66).

## Conclusion

The new method allows determining the efficacy of hand rubs against MNV. The contamination is clinically relevant, the application of a hand rub is done as in patient care, the negative control allows determining the reproducibility.

## Disclosure of interest

G. Kampf Employee of Bode Chemie GmbH, Hamburg, Germany, E. Steinmann Consultant for Mikrolab GmbH, Bremen, Germany, J. Steinmann Employee of Mikrolab GmbH, Bremen, Germany.

